# Interaction of Perfluorooctanoate and Perfluorohexanoate with Seed Protein

**DOI:** 10.1021/acs.langmuir.5c01879

**Published:** 2025-06-20

**Authors:** Njelama Sanga, Nicholas Croy, Lutz Ahrens, Rebecca J. L. Welbourn, Adrian R. Rennie

**Affiliations:** 1 Macromolecular Chemistry, Department of Chemistry - Ångström, 8097 Uppsala University, Box 539, Uppsala 75120, Sweden; 2 Molecular Biomimetics, Department of Chemistry - Ångström, 8097 Uppsala University, Box 523, Uppsala 75120, Sweden; 3 Department of Aquatic Sciences and Assessment, Swedish University of Agricultural Sciences (SLU), Box 7050 Uppsala SE-750 07, Sweden; 4 Rutherford Appleton Laboratory, Harwell, Didcot OX11 0QX, United Kingdom

## Abstract

Per- and polyfluoroalkyl substances (PFAS) are persistent and potentially toxic pollutants found widely in the environment; however, there is a lack of understanding how these materials interact with many interfaces that are important for remediation. The association of perfluorooctanoate (PFOA) and perfluorohexanoate (PFHxA) with seed protein was investigated using neutron reflectometry. The seed protein is known to associate with many materials and adsorbs irreversibly to silica surfaces, and it was shown that it was not removed by rinsing with water. PFHxA and PFOA were found to adsorb to the previously bound protein, forming mixed layers of protein, surfactant, and water that expanded to incorporate the extra material. On rinsing with water, PFOA was removed from the layer, leaving the protein bound to the silica surface. An almost three-times larger volume fraction of PFOA than PFHxA was observed in the protein layer. At the critical micelle concentration, the layer consisted of 1.8 mg m^–2^ PFOA and 1.3 mg m^–2^ of protein. Comparison of the relative amounts of each surfactant and protein suggests that hydrophobic interactions play a significant role in the coadsorption. The results indicate that the seed protein could be used to adsorb PFAS reversibly as a step toward remediation of contamination. This quantification of association with an albumin-like protein is important for understanding of transport both in human bodies and in the environment.

## Graphical abstract

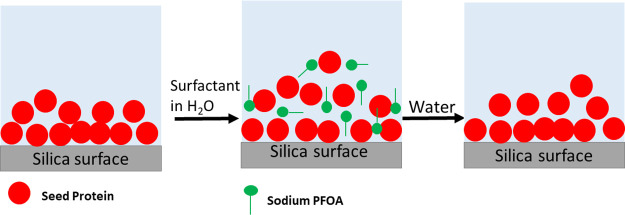

## 1 Introduction

Per- and polyfluoroalkyl substances (PFAS) are widely used across various industries due to their unique properties. Their chemical stability, along with their hydrophobic and oleophobic nature, allows them to be used in applications such as fire-fighting foams, protective coatings, and cookware.^1^ However, their properties make PFAS bioaccumulative and they are recognized as hazardous due to their toxic and persistent nature.^2,3^ A subset of PFAS, perfluoroalkyl carboxylates, are surfactants. There is interest in understanding how these amphiphiles such as perfluorooctanoate (PFOA) and perfluorohexanoate (PFHxA) interact with materials in the environment like minerals, inorganic salts, and natural organic matter such as seed proteins. Previous studies on PFOA have shown how they adsorb to interfaces such as alumina but not silica that is anionic.^4^ They self-assemble as micelles^5^ and adsorb to sediments such as clay particles.^6^ A study observed that PFHxA associated with, and penetrated, a supported phospholipid bilayer on a crystal surface, displacing the lipid.^7^ Understanding the complexity of these interactions and absorption mechanisms of these surfactants with materials is important for applications, such as remediation.

Seed protein from trees has attracted attention not just as a food source but as a material that can be used to flocculate contaminants in water.^8–10^ The use as a flocculating agent has been shown to be effective because the seed protein adsorbs to many different interfaces and tends to self-associate. Studies have also been made of the selective binding of water-soluble contaminants such as heavy metal ions to the seed material.^11,12^ Recent interest has been shown in its use for remediation of PFAS as the seed material is rich in 2*S*-albumin.^13^ A further motivation for studies is that there is similarity to serum albumins found in the blood, which have shown to bind and transport PFAS efficiently.^14–16^ Several studies have examined the interactions of seed proteins with various classes of surfactants.^17–20^ The proteins are cationic with an isoelectric point above pH 10 and zeta potential at a neutral pH of 14 ± 2 mV.^19^ These studies show that the protein associates strongly with the surfactants, and the interactions were dominated by electrostatic attraction and some hydrophobic interactions. Understanding the interactions offers future prospects for designing various applications that can be used for remediation strategies. For example, a suggested application has been to use sand precoated with a layer of protein as an antimicrobial filter for bacteria^21^ and the regeneration of such filters using dodecyl glucoside and sodium dodecyl sulfate was investigated.^22^ Dodecyl glucoside removed the bacteria without displacing the adsorbed protein, whereas the sodium dodecyl sulfate displaced the protein on the sand filters. Another approach exploiting the capability to bind pollutants has been to encapsulate the seed protein with biochar in alginate beads. Previous studies have investigated whether perfluorooctanesulfonate (PFOS) and perfluorobutanesulfonate (PFBS) were adsorbed by these beads. The ability to bind to small amounts of these surfactants was identified.^23,24^

In the present work, neutron reflectometry is used to investigate the interaction of the fluorocarbon surfactants sodium PFOA and sodium PFHxA with layers of the seed protein adsorbed at a solid/solution interface. Reviews^25,26^ describe the principles and how the technique can be used to study interfaces by exploiting isotopes of hydrogen to change contrast and highlight individual components in mixtures. The study investigated whether sodium PFOA and sodium PFHxA adsorb to the protein. Multiple contrasts of water (D_2_O and H_2_O) were used to determine the composition and structure of the mixed layer. Further, the amounts of the surfactant bound were quantified, and the reversibility of its binding to the protein was tested.

## 2 Materials and Methods

### 2.1 Perfluorinated Surfactants (PFAS) and Adsorption Substrates

Sodium PFOA and sodium PFHxA were prepared in the laboratory by neutralization of perfluorooctanoic acid (PFOA) purchased from Sigma-Aldrich (C_7_F_15_COOH, purity:95%) and PFHxA purchased from Sigma-Aldrich (C_5_F_11_COOH, purity: 97%) with sodium hydroxide purchased from Sigma-Aldrich (NaOH, purity: 98%). The solution was stirred until a homogeneous solution was formed. The neutralized solutions were evaporated to dryness in an oven at 60 °C. For the neutron experiments, H_2_O was obtained from a Millipore system and D_2_O (99.8% D) was supplied by Thermo Scientific. The seed protein was extracted from seeds from Livingstone, Zambia and used in previous studies.^27^ The Supporting Information (Supporting Information) describes how the surfactant and protein solutions were prepared. The adsorption substrates were silicon crystals purchased from Crystran cut to expose the (111) crystal face and polished to a low roughness. The surface was cleaned with concentrated sulfuric acid according to the procedure reported by Nouhi et al.^28,29^ Measurements were made with solution concentrations up to the critical micelle concentrations that were estimated as 18 and 110 mmol dm^–3^ from conductivity measurements for sodium PFOA and sodium PFHxA, respectively.

### 2.2 Neutron Reflection Experiments

The neutron reflection experiments were performed on the Offspec^30^ instrument at the ISIS Pulsed Neutron and Muon Source, Rutherford Appleton Laboratory, Didcot, United Kingdom. Data were recorded at angles of 0.6, 1.2, and 2.3° with wavelengths from 1.5 to 14.5 Å and the resulting reflectivity profiles combined to provide a momentum transfer, *Q*, between 0.01 and 0.25 Å^–1^ with a resolution of 3% Δ*Q*/*Q*. The reflection substrates were mounted with a flow-through sample cell^31^ with a volume of about 2.5 mL. All measurements were made at 25 °C using a Julabo water bath circulated through the cell housing. Sample solutions were injected manually into the cell, and a Knauer HPLC pump was used to provide H_2_O and D_2_O in the required mixing ratios.

The experiment utilized four different water contrasts: 100% D_2_O, 63%/37% D_2_O/H_2_O (fluorocarbon matched water, FMW), 38%/62% D_2_O/H_2_O (silicon matched water, SMW), and 100% H_2_O. The fluorocarbon matched water was measured at an additional angle of 0.35° due to its low critical edge.

### Interpretation of Neutron Reflection Data

Neutron reflectivity is the ratio of the intensity of the reflected beam to the incident beam. This varies with angle and wavelength as a function of the momentum transfer, *Q* = (4π/λ) sin­(θ_*i*_), where λ is the wavelength of the neutrons and θ_*i*_ is the angle of incidence. These data can be fitted to models for the interface structure. Neutrons are sensitive to different isotopes such as hydrogen and deuterium, and this allows modifying the refractive index without changes to the chemical composition. This enables the determination of detailed information about the composition and structure. In particular, the use of mixtures of H_2_O and D_2_O in different ratios, to match the contrast of one component in a mixed system, can highlight the features of other components. In this way, neutron reflectivity can determine the amount of each component in a layer at interfaces as well as the thickness. The reflection signal depends on the refractive index differences between the layers that form an interface.^32^ The refractive index, *n*, for the neutrons is related to the scattering length density, ρ, of the material by *n* = 1–(λ^2^/2π)­ρ. The scattering length density is the sum of the scattering lengths, *b_i_*, of the atoms divided by their volume, *V*, and is calculated as ρ = Σ*n_i_b_i_*/*V* where *n_i_* is the number density of atoms of element *i*, and the sum is taken over all the elements in the layer. The scattering length has been measured experimentally and tabulated for the different elements and isotopes.^33^ The molecular formulas and scattering lengths and scattering length densities of the materials used in this study are listed in Table 1. The two scattering length densities of the protein account for the exchange of protons with the water.

**Table 1:** Materials and Chemicals Used with Neutron Scattering Lengths and Scattering Length Densities

name	formula	formula mass (g mol^–1^)	volume (Å^3^)	scattering length Σb/fm	ρ (10^–6^ Å^–2^)
water	H_2_O	18	30.0	–1.67	–0.56
deuterated water	D_2_O	20	30.0	19	6.35
silicon	Si	28	20.0	4.15	2.07
silica	SiO_2_	60	45.7	15.7	3.4
sodium PFOA	C_8_F_15_O_2_Na	436	409	156	3.81
sodium PFHxA	C_6_F_11_O_2_Na	336	310	119	3.85
protein in H_2_O		7307	9120	1312	1.44
protein in D_2_O		7385	9120	2337	2.56

Data for adsorbed layers were modeled by making a combined fit of the different contrasts to structures that consisted of layers at the interface. An inner layer was used to model the oxide at the surface of the silicon crystal. Parameters for the substrate were constrained to those found for the initial measurements on clean surfaces. For the bound protein and protein with PFAS, the model included a further single layer of uniform composition and a profile decaying exponentially to the bulk solution density. These are parametrized by thickness, *t*, scattering length density, and the exponential decay length, *l*. The volume fraction of the protein layer with a fixed concentration, Φ_p_, is calculated from the known scattering length densities of the materials. In the presence of surfactants, a similar model provided good fits, with both surfactant and protein concentration decreasing away from the surface. Acceptable structural models were constrained by the requirement that the same concentrations of protein and, when appropriate, surfactant fitted all of the isotopic contrasts. The models were made using the software for profiles of density at interfaces described previously.^17,18^ In all cases, 50 steps were used to represent the exponential decay.

The amount of material bound to the interface, the surface excess of the protein, Γ_p_, and the surfactant, Γ_s_, are readily calculated by integrating the volume fraction profiles. For the model with a uniform layer and an exponential decay, these are simple given by Γ_p_ = Φ_p_(*t* + *l*)­ρ_p_ and Γ_s_ = Φ_s_(*t* + *l*)­ρ_s_, where ρ_p_ and ρ_s_ are the mass densities of protein and surfactant, respectively.

## 3 Results and Discussion

### 3.1 Binding of Protein to Silica

At the beginning of the experiment, the reflection surfaces (silica 1 and silica 2) were characterized in these water contrasts to determine the thickness and roughness of the surface oxide layer. The results are shown in Figures S1 and S2 with the parameters in Table S1 (Supporting Information). After this characterization, the two surfaces were exposed to 0.15 wt.% protein in D_2_O. The neutron reflectivity from the adsorbed layer of protein to silica 1 is shown in Figure S3 in the Supporting Information. After exposure of the surface to protein, it was measured in three contrasts of water (D_2_O, H_2_O, and fluorocarbon matched water (FMW)). It was verified that, as previously,^17,18^ the rinsing did not displace protein by checking the similarity of the reflectivity in the presence of protein solution and with pure D_2_O. The combined data for all contrasts for the layer on silica 1 were fitted to a model that included the 17 ± 1 Å oxide layer and the adsorbed protein that consisted of a uniform layer of protein and water with a thickness, *t*, of 6 ± 1 Å with a volume fraction of 0.46 that decays toward the bulk of the solution with an exponential decay length of 15 ± 1 Å. The model corresponds to an adsorbed amount of protein of 1.3 ± 0.1 mg m^–2^ (or 0.20 ± 0.02 μmol m^–2^). On silica 2, the neutron reflectivity data shown in Figure S4 in the Supporting Information was fitted to a similar structure but with more protein bound with a layer of 14 Å and a volume fraction of 0.48 that decays with a 31 Å decay length that was twice of silica 1.

### 3.2 Interactions of Sodium PFOA with Protein

Measurements were made to determine how 18 mmol dm^–3^ sodium PFOA solutions interacted with a preadsorbed layer of protein on silica 1. Clear differences in the reflectivity were seen when the surfactant solution was injected. The details of the structure and composition of the interface with both protein and surfactant could be measured with solutions in different water contrasts at the same concentration in contact with the protein layer at the interface.

The neutron reflectivity data for solutions of 18 mmol dm^–3^ sodium PFOA in contact with the preadsorbed layer of on silica 1 for the three water contrasts are shown in Figure 1. The data were fitted with the assumption that a constant amount of protein remained on the surface, as it was observed that when the surfactant solution was replaced by rinsing with pure water, the reflectivity reverted to that of the surface with just protein, as seen in Figure 2. The hydrated protein layers expand to accommodate the sodium PFOA. The presence of the surfactant tended to increase the overall thickness. Simultaneous fits to all contrasts of surfactant solution allowed calculation of the amount of adsorbed sodium PFOA and protein in the layer. The fit indicated that the region of constant composition expanded to a thickness of 22 Å with the exponential decrease of concentration having a decay length of 19 Å. This corresponds to an amount of surfactant that is mixed in the layer of 1. 8 ± 0.1 mg m^–2^ or 4.1 ± 0.1 μmol m^–2^.

**Figure 1:**
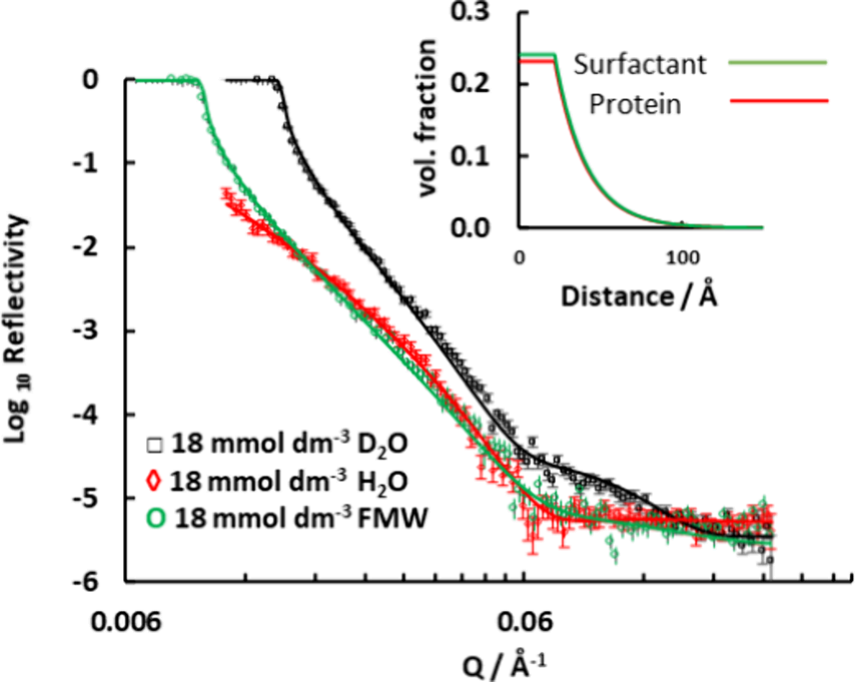
Neutron reflectivity data for 18 mmol dm^–3^ sodium PFOA to a preadsorbed layer of protein (1.3 mg m^–2^) on silica in three different water contrasts. The three contrasts allow direct calculation of the amount of adsorbed sodium PFOA in the layer, which also contains protein and water. The solid lines show a model that fits simultaneously each contrast with a uniformly mixed layer of 23 Å that decays toward the bulk solution with an exponential decay length of 19 Å. The clear difference of this data to that for the layers of protein alone is seen in Figure S5 in the Supporting Information. The inset shows the volume fraction density profile for and sodium PFOA in the layer.

**Figure 2:**
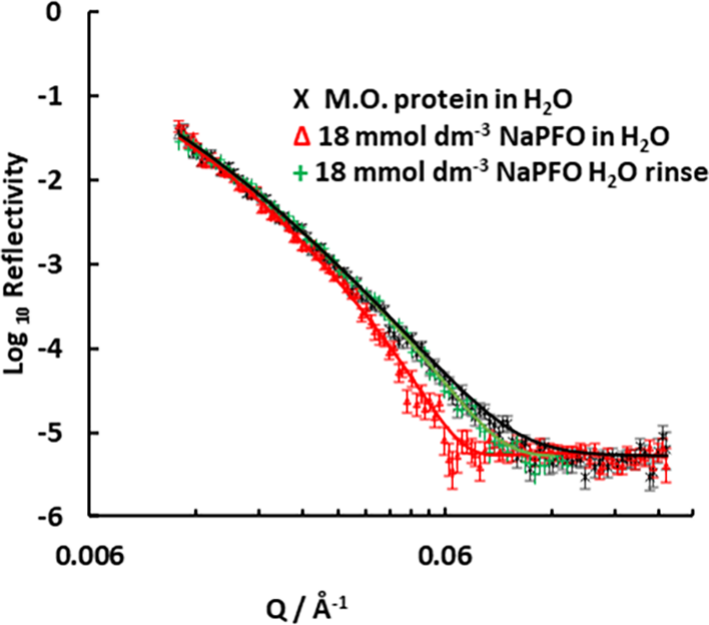
Change in neutron reflectivity data after rinsing adsorbed sodium PFOA (from Figure 1) on the preadsorbed protein solutions in H_2_O. The lines are the model fits for protein alone, for protein with 18 mmol dm^–3^ sodium PFOA, and after the rinse with water.

### 3.3 Effect of Rinsing with Water

Measurements were taken to determine whether the surfactant had adsorbed to the protein reversibly. The surface was rinsed after exposure to 18 mmol dm^–3^ sodium PFOA and the measured reflectivity in Figure 2 shows that the surfactant was removed while the protein remained on the surface. Rinsing with H_2_O causes the measured data to almost overlap with the initial measurement for protein adsorbed to the surface. The water removed the surfactant, but the amount of protein on the surface remained almost constant.

### 3.4 Effect of Sodium PFOA Concentration on the Adsorption to Moringa Seed Protein

To investigate the influence of concentration, solutions of sodium PFOA in H_2_O at 4.5, 9, and 18 mmol dm^–3^ were measured in contact with the preadsorbed protein on silica 1. The data in Figure 3 shows that for successively higher concentrations, the curves tend toward overlap at a critical micelle concentration of 18 mmol dm^–3^. The highest concentration was investigated further with additional contrasts to provide detail and structure of the full plateau coverage as has been described in Section 3.2. Plots of the reflectivity data for the lower concentrations are shown in Figure S8 in the Supporting Information. Under the assumption that the structural model described previously for the results at the critical micelle concentration can be used, the surface excess of the surfactant could be calculated from the model fits and is shown as an inset in Figure 3. It is seen to vary only weakly in the range of concentrations that were investigated.

**Figure 3:**
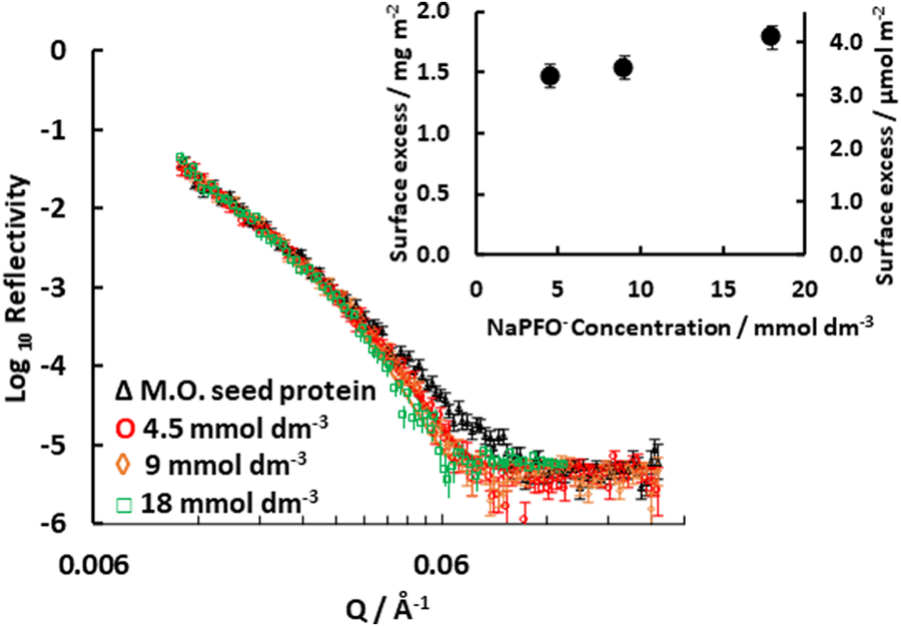
Changes in neutron reflectivity for sodium PFOA at different concentrations added to the preadsorbed layer of seed protein for solutions in H_2_O. The inset shows the surface excess of sodium PFOA adsorbed at the various concentrations up to the critical micelle concentration. The lines show the fitted model with the parameters in Table S3.

### 3.5 Interactions of Sodium PFHxA with Protein

A similar series of experiments were performed to compare the behavior of the smaller surfactant, sodium PFHxA, with the layer of protein on silica 2. Figure 4 shows the change in neutron reflectivity on exposing the preadsorbed layer to this surfactant at the critical micelle concentration, 110 mmol dm^–3^. This substrate had twice the amount of preadsorbed protein as compared to silica 1. It is likely that the variation in the adsorbed amount could have arisen as a consequence of different displacement of the water from the cell during manual injection of the protein solutions. Sodium PFHxA also interacts with the protein, forming a mixed layer with protein, surfactant, and water. The solid lines show a model fit for a layer of constant composition with a thickness of 55 Å that decays with a characteristic length of 37 Å. The amount of sodium PFHxA in the layer is 1.3 ± 0.1 mg mm^–2^ or 4.1 ± 0.1 μmol m^–2^.

**Figure 4:**
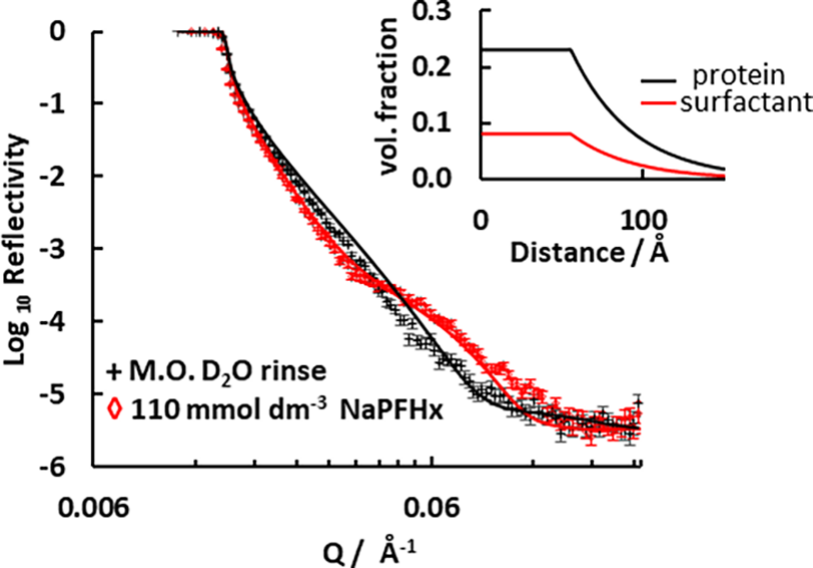
Change in neutron reflectivity on exposing a preadsorbed layer of seed protein, to 110 mmol dm^–3^ sodium PFHxA solution in D_2_O. The lines are the model fits for protein alone and for protein with 110 mmol dm^–3^ sodium PFHxA. The inset shows the density profile of in the mixed uniform layer.

As reported in previous studies^18,28^ the protein binds to silica/solution interfaces to form an adsorbed layer that is not removed by rinsing with water. The structure of the adsorbed protein consisted of a thin layer near the surface that contained approximately equal volumes of protein and water with a profile that decayed smoothly to pure solvent. The amount of protein depended slightly on the initial concentration of the solution and thus influenced the thickness. The sodium PFOA data were fitted with a similar model with a scattering length density corresponding to a mixture of surfactant and protein for multiple contrasts, and this implies that there was no separation in the layer with sodium PFOA uniformly mixed. Near the silica substrate, the layer consists of a volume fraction of 23% sodium PFOA, 24% protein, and 53% water. In contrast, sodium PFHxA formed a mixed layer of volume fraction of 8% sodium PFHxA, 23% protein, and 69% water. The volume fraction of sodium PFHxA in the mixed layer is three-times lower than that of sodium PFOA. The molar ratio of sodium PFHxA to protein is 10 compared with 23 for sodium PFOA. It is interesting to note that the sodium PFHxA binds to a significantly greater extent than a hydrocarbon surfactant, sodium dodecyl sulfate, that was observed by measurements of zeta potential to neutralize the charge on a protein molecule in solution with about seven surfactant molecules.^19^ The large difference in the amount of bound sodium PFHxA to that of the sodium PFOA suggests that the binding is not simply due to the anionic surfactant head groups interacting with the cationic moieties of the protein and that hydrophobic interactions play a role in the association.

It has been reported previously that anionic protein associates with the anionic surfactant sodium dodecyl sulfate in solution.^17,18^ Zeta potential measurements^19^ on mixed solutions of protein with sodium dodecyl sulfate showed binding changing with concentration of surfactant. The decrease of the zeta potential to negative values as the concentration increased indicated hydrophobic association of the surfactant with the protein.^19^ At the solid interface, sodium dodecyl sulfate adsorbed reversibly like sodium PFOA in this study and was removed from the layer by rinsing.^17,18^ Hexadecyltrimethylammonium bromide (CTAB), a cationic surfactant, however, was observed to displace the protein layer from an alumina interface.^17^ PFOA on its own was observed to adsorb to alumina surfaces, not silica. The alumina, in contrast to silica, has a positive surface potential at neutral pH and the adsorption of anionic surfactants is favored by electrostatic interactions.^4^

Other studies have examined the interactions of PFOS and PFBS with seed material encapsulated in alginate beads at low concentrations.^23,24^ The binding reached an adsorption limit of surfactant of less than 1 mg g^–1^ adsorbent^23^ for PFOS, and this amount was about a factor of 30 less than that seen in the present study with purified protein. This suggests that an improved process for removal or remediation might be achieved at least for some PFAS, e.g., PFOA and PFOS, where, if necessary, the seed protein could be supported on silica or other mineral substrates. Further studies would be needed to determine whether the functional group (PFOA vs PFOS) affects the binding to the seed protein.

It is interesting to note that a different study of the potential of seed extract for remediation of solutions of surfactants used sodium dodecyl sulfate at concentrations below the critical micelle concentration.^34^ In this case, binding reached a limit of approximately 0.6 g of surfactant per 1 g of seed material, suggesting a limiting composition of around 40% by weight of seed material. This would represent approximately similar volume composition to those found for sodium PFOA and sodium PFHxA in the present study, although the protein/surfactant complexes are significantly hydrated. The interactions of sodium dodecyl sulfate were found to be at least partially due to hydrophobic association as well as attraction between the anionic surfactant and the cationic protein.^34^ These interactions are analogous to those suggested in the present study.

The quantitative knowledge about binding of PFAS to an albumin-like protein is useful as this process is an important transport pathway of PFAS in organisms^14–16^ that is related to eventual bioaccumulation. The reversible association can allow the surfactants to move readily with the blood plasma before they are released from albumin when they encounter preferential binding sites such as the liver. It has been suggested^35^ that the surfactant associates with a fatty acid binding protein. Unlike the reversible interaction with the albumin-like proteins, the binding of PFHxA and PFOA to the components in the liver is likely not easily reversible due to stronger interactions.

## 4 Conclusions

To summarize, it has been shown that 1.8 mg m^–2^ sodium PFOA and 1.3 mg m^–2^ sodium PFHxA bound to seed protein adsorbed on a silica surface. Model fits of the structure from neutron reflectometry experiments show that the surfactants penetrated the hydrated protein layer, which expanded to accommodate it rather than forming a separate outer layer on top. This suggests that the interaction between the surfactant and seed protein was not just due to the neutralization of charges but also arose from hydrophobic interactions. The present study shows that sodium PFOA interacts with a much higher affinity for the seed protein than that reported for another fluorocarbon surfactant, PFOS, with the protein encapsulated in alginate beads.^23^ The binding per unit mass of protein identified in the present study was about a factor of 30 higher than seen in the previous work. The study indicates a route toward practical applications for removal of PFAS using protein. The adsorption of PFOA was readily reversed by rinsing with pure water. In contrast the protein remains bound to the supporting silica. This provides a practical solution for regeneration and reuse of the protein during the treatment process.

## Supplementary Material



## Notes

Plots of reflectometry data with model fits for substrates and substrates with seed protein; tables of fit parameters and details of solution preparation (PDF)

## References

[1] Buck R. C., Franklin J., Berger U., Conder J. M., Cousins I. T., De Voogt P., Jensen A. A., Kannan K., Mabury S. A., Van Leeuwen S. P. J. (2011). Perfluoroalkyl and Polyfluoroalkyl Substances in the
Environment: Terminology, Classification, and Origins. Integr. Environ. Assess Manag.

[2] Ahrens L. (2011). Polyfluoroalkyl
Compounds in the Aquatic Environment: A Review of their Occurrence
and Fate. J. Environ. Monit..

[3] Abunada Z., Alazaiza M. Y. D., Bashir M. J. K. (2020). An Overview
of Per-and Polyfluoroalkyl
Substances (PFAS) in the Environment: Source, Fate, Risk and Regulations. Water.

[4] Hellsing M. S., Josefsson S., Hughes A. V., Ahrens L. (2016). Sorption of Perfluoroalkyl
Substances to Two Types of Minerals. Chemosphere.

[5] López-Fontán J. L., Sarmiento F., Schulz P. C. (2005). The Aggregation of Sodium Perfluorooctanoate
in Water. Colloid Polym. Sci..

[6] Jeon J., Kannan K., Lim B. J., An K. G., Kim S. D. (2011). Effects
of Salinity and Organic Matter on the Partitioning of Perfluoroalkyl
Acid (PFAs) to Clay Particles. J. Environ. Monit..

[7] Nouhi S., Ahrens L., Pereira H. C., Hughes A. V., Campana M., Gutfreund P., Palsson G. K., Vorobiev A., Hellsing M. S. (2018). Interactions
of Perfluoroalkyl Substances with a Phospholipid Bilayer studied by
Neutron Reflectometry. J. Colloid Interface
Sci..

[8] Kansal S. K., Kumari A. (2014). Potential of *M. oleifera* for the Treatment
of Water and Wastewater. Chem. Rev..

[9] Herrera J. C. G., Ovallos C. A. M., Agudelo-Castaneda D. M., Partenina-Arbodela C. D. (2024). Exploring *Moringa oleifera*: Green Solutions for Sustainable Wastewater Treatment and Agricultural
Advancement. Sustainability.

[10] Ndabigengesere A., Narasiah K. S. (1998). Quality of Water Treated by Coagulation using *Moringa oleifera* seeds. Wat. Res..

[11] Gomes H. d. O., Freire P. d. T. C., do Nascimento R. F., Pereira Teixeira R. N. (2022). Removal of Contaminants from Water
using Moringa oleifera
Lam. as Biosorbent: An overview of the Last Decade, Water. Proc. Eng..

[12] Alghamdi A., Rajan K. P., Thomas S. P. (2024). Comprehensive
Evaluation of *Moringa oleifera* Seed as a Low-cost
Adsorbent for Removal
of Manganese (Mn) from Aqueous Solutions. Chem.
Env. Eng..

[13] Moulin M., Mossou E., Signor L., Kieffer-Jaquinod S., Kwaambwa H. M., Nermark F., Gutfreund P., Mitchell E. P., Haertlein M., Forsyth V. T., Rennie A. R. (2019). Towards
a Molecular understanding of the Water Purification Properties of *Moringa* Seed Proteins. J. Colloid
Interface Sci..

[14] Bangma J., Guillette T. C., Bommarito P. A., Ng C., Reiner J. L., Lindstrom A. B., Strynar M. J. (2022). Understanding the
Dynamics of Physiological
Changes, Protein Expression, and PFAS in Wildlife. Environ. Inter..

[15] Forsthuber M., Kaiser A. M., Granitzer S., Hassl I., Hengstschläger M., Stangl H., Gundacker C. (2020). Albumin is the Major Carrier Protein
for PFOS, PFOA, PFHxS, PFNA and PFDA in Human Plasma. Environ. Int..

[16] Pye E. S., Wallace S. E., Marangoni D. G., Foo A. C. Y. (2023). Albumin Proteins
as Delivery Vehicles for PFAS Contaminants in Respiratory Membranes. ACS Omega.

[17] Kwaambwa H. M., Hellsing M. S., Rennie A. R., Barker R. (2015). Interaction
of *Moringa oleifera* Seed Protein with a Mineral Surface
and
the Influence of Surfactants. J. Colloid Interface
Sci..

[18] Kwaambwa H. M., Hellsing M. S., Rennie A. R. (2010). Adsorption of a Water Treatment Protein
from *Moringa oleifera* Seeds to a Silicon Oxide Surface
Studied by Neutron Reflection. Langmuir.

[19] Kwaambwa H. M., Rennie A. R. (2012). Interactions of
Surfactants with a Water Treatment
Protein from *Moringa Oleifera* Seeds in Solution Studied
by Zeta-Potential and Light Scattering Measurements. Biopolymers.

[20] Nouhi S., Pascual M., Hellsing M. S., Kwaambwa H. M., Skoda M. W. A., Höök F., Rennie A. R. (2018). Sticking Particles
to Solid Surfaces using *Moringa oleifera* Proteins
as a Glue. Colloids Surf., B.

[21] Jerri H. A., Adolfsen K. J., McCullough L. R., Velegol D., Velegol S. B. (2012). Antimicrobial
Sand via Adsorption of Cationic *Moringa Oleifera* Protein. Langmuir.

[22] Williams F. E., Lee A. K., Orandi S., Sims A. K., Lewis D. M. (2017). Moringa
oleifera Functionalised Sand-Reuse with Non-Ionic Surfactant Dodecyl
Glucoside. J. Water Health.

[23] Militao I. M., Roddick F., Fan L., Zepeda L. C., Parthasarathy R., Bergamasco R. (2023). PFAS Removal
from Water by Adsorption with Alginate-Encapsulated
Plant Albumin and Rice Straw-Derived Biochar. Journal of Water Process Engineering.

[24] Militao I. M., Roddick F., Bergamasco R., Fan L. (2022). Rapid Adsorption of
PFAS: Application of *Moringa oleifera* Seed Powder
Encapsulated in Alginate Beads. Environ. Technol.
& Innovation.

[25] Penfold J., Thomas R. K. (1990). The Application of the Specular Reflection
of Neutrons
to the Study of Surfaces and Interfaces. J.
Phys: Condens. Matter.

[26] Lu J. R., Thomas R. K., Penfold J. (2000). Surfactant Layers at
the Air/Water
Interface: Structure and Composition. Adv. Colloid.
Interface Sci..

[27] Maikokera R., Kwaambwa H. M. (2007). Interfacial properties and fluorescence
of a coagulating
protein extracted from *Moringa Oleifera* seeds and
its interaction with sodium dodecyl sulphate. Colloids and Surf. B: Biointerfaces.

[28] Nouhi S., Kwaambwa H. M., Gutfreund P., Rennie A. R. (2019). Comparative Study
of Flocculation and Adsorption Behaviour of Water Treatment Proteins
from *Moringa peregrina* and *Moringa oleifera* seeds. Sci. Rep..

[29] Nouhi S., Ahrens L., Campos Pereira H., Hughes A. V., Campana M., Gutfreund P., Palsson G. K., Vorobiev A., Hellsing M. S. (2018). Interactions
of Perfluoroalkyl Substances with a Phospholipid Bilayer studied by
Neutron Reflectometry. J. Colloid Interface
Sci..

[30] Webster J. R. P., Langridge S., Dalgliesh R. M., Charlton T. R. (2011). Reflectometry techniques
on the Second Target Station at ISIS: Methods and Science. Eur. Phys. J. Plus.

[31] Rennie A. R., Hellsing M. S., Lindholm E., Olsson A. (2015). Note: Sample Cells
to Investigate Solid/Liquid Interfaces with Neutrons. Rev. Sci. Instrum..

[32] Fermon, C. ; Menelle, A. ; Neutron Reflectometry, In: Daillant, J. ; Gibaud, A. ;(eds) X-ray and Neutron Reflectivity: Lect. Notes in Phys. 770, 2009, Springer: Berlin Heidelberg.

[33] Sears V. (1992). F, Neutron
Scattering Lengths and Cross sections. Neutron
News.

[34] Beltrán-Heredia J., Sánchez-Martín J. (2009). Removal of
Sodium Lauryl Sulphate
by Coagulation/Flocculation with *Moringa oleifera* Seed Extract. Journal of Hazardous Materials..

[35] Zhang L., Ren X.-M., Guo L.-H. (2013). Structure-based
Investigation on
the Interaction of Perfluorinated Compounds with Human Liver Fatty
Acid Binding Protein. Environ. Sci. Technol..

