# Supplementary material for: Interaction of Perfluorooctanoate and Perfluorohexanoate with Seed Protein
Source: Langmuir. 2025 Jun 20;41(25):16500–5. doi: 10.1021/acs.langmuir.5c01879 (PMC12224311; doi:10.1021/acs.langmuir.5c01879)
Supplement: Supplementary file 1 [file la5c01879_si_001.pdf]

Supporting information

Interaction of perfluorooctanoate and perfluorohexanoate with Moringa oleifera seed protein

Njelama Sanga[1], Nicholas Croy[2], Lutz Ahrens[3], Rebecca J. L. Welbourn[4], Adrian R. Rennie[1]

[1] Macromolecular Chemistry, Department of Chemistry - Ångström, Uppsala University, Box 539, 75120, Uppsala, Sweden

[2] Molecular Biomimetics, Department of Chemistry - Ångström, Uppsala University, Box 523, 75120 Uppsala, Sweden

[3] Department of Aquatic Sciences and Assessment, Swedish University of Agricultural Sciences, Box 7050 Uppsala, Sweden

[4] Rutherford Appleton Laboratory, Harwell, Didcot OX11 0QX, United Kingdom - Present Address: Oak Ridge National Laboratory, Oak Ridge, TN 37832, USA.

Preparation of solutions

The protein stock solution was prepared at 0.15 wt. % in D_2O and tumbled overnight. The pH of the solution was 6. The sodium perfluorooctanoate stock solution was prepared at 18 mmol dm^{-3} by directly dissolving the powder in four different water contrasts H_2O, D_2O, 38%$_{vol}$ D_2O 62%$_{vol}$ H_2O, 63%$_{vol}$ D_2O: 37%$_{vol}$ H_2O. Dilutions of the 18 mmol dm^{-3} stock solution in H_2O to 4.5 mmol dm^{-3} and 9 mmol dm^{-3}, respectively, were prepared by weight. The stock solutions of sodium perfluorohexanoate were prepared at 110 mmol dm^{-3} in the same water contrasts as above. The pH of both surfactant solutions was 7.

Preparation of Substrates

The substrates were cleaned prior to the experiment by putting them in diluted Decon90 in a clean petri dish. The substrates were then washed with pure water. The cleaning continued by spreading a few drops of concentrated sulfuric acid over the surface of the substrate and adding the same amount of water. After leaving for five minutes, the substrate was rinsed with an excess of pure water. The substrate was cleaned with the acid two more times or until the surface of the crystal was uniformly hydrophilic with no contact angle for water.

Measurement Protocol

After characterization of the surfaces, 5 mL of 0.15 wt.% protein solution in D_2O was manually injected into both the sample cells so as to displace the D_2O already in the cell and the neutron reflectivity was measured. After these measurements, 30 mL D_2O was pumped into the sample cell at a flow rate of 1 mL min^{-1}. The reflectivity was measured again to determine if the protein was displaced. Then, 30 mL H_2O was pumped into the sample cell at a flow rate of 1 mL min^{-1}. An additional measurement was taken to check again if the protein was displaced by rinsing in another contrast. The surfactant was then introduced by manually injecting 10 mL of the surfactant in water. On the silica 1 surface, a series of increasing concentrations were manually injected (10 mL) and neutron reflectivity was measured. After, 30 mL of H_2O was pumped to identify what was removed by rinsing.

Characterization of silica surfaces 1 and 2

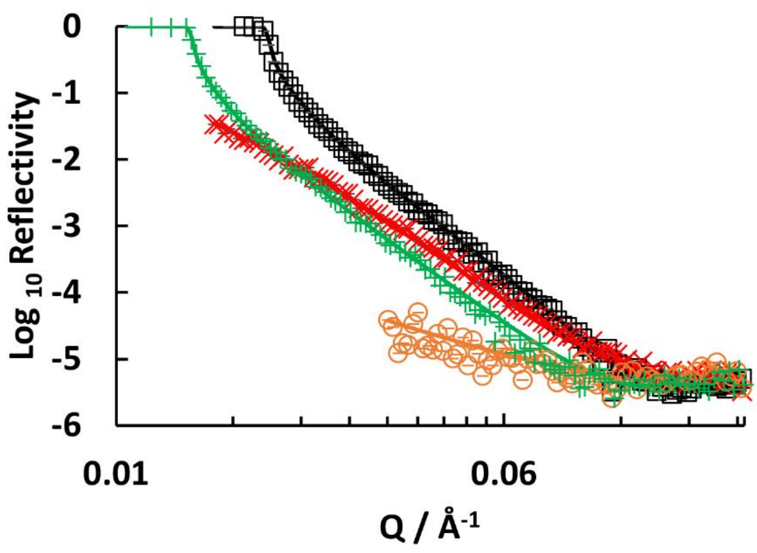

Figure S1. Reflectivity curves for characterization of the oxide layer on silicon in (□) D_2O, (+) fluorocarbon matched water, (o) Si match, and (x) H_2O for the substrate silica 1. The solid lines show the fitted model with parameters given in Table S1.

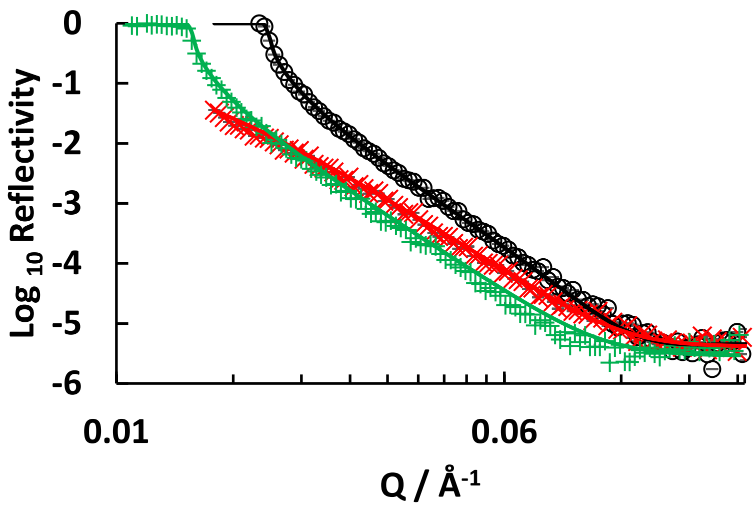

Figure S2. Reflectivity curves for the characterization of the oxide layer on silicon in (o) D_2O, (+) fluorocarbon matched water, and (x) H_2O on for the substrate silica 2. The solid lines show the fitted model with parameters given in Table S1.

Table S1. Fitted parameters for the characterization of the substrates

	t_p / Å	Water hydration %	Roughness / Å
Silica 1	17 ± 1	38 ± 1	4 ±1
Silica 2	17 ± 1	37 ± 1	4 ± 1

Characterization of *Moringa oleifera* seed protein on Silica 1 and Silica 2

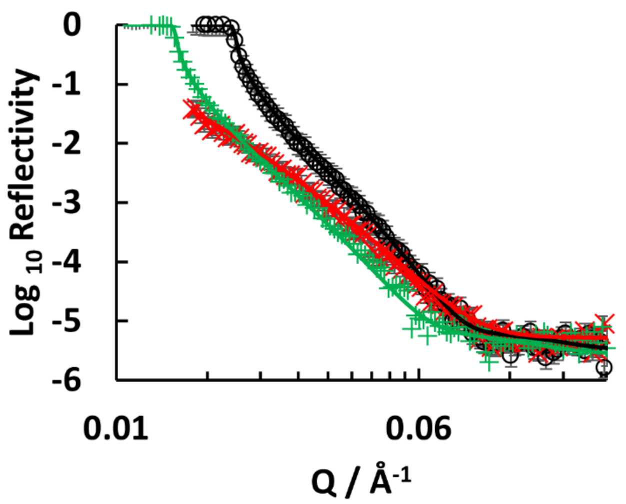

Figure S3. Neutron reflectivity data showing the addition of 0.15 wt. % *Moringa oleifera* seed protein to the cleaned silica one surface in (o) D_2O, (+) fluorocarbon matched water, and (x) H_2O. The solid lines show a model fit with parameters that are shown in Table S4.

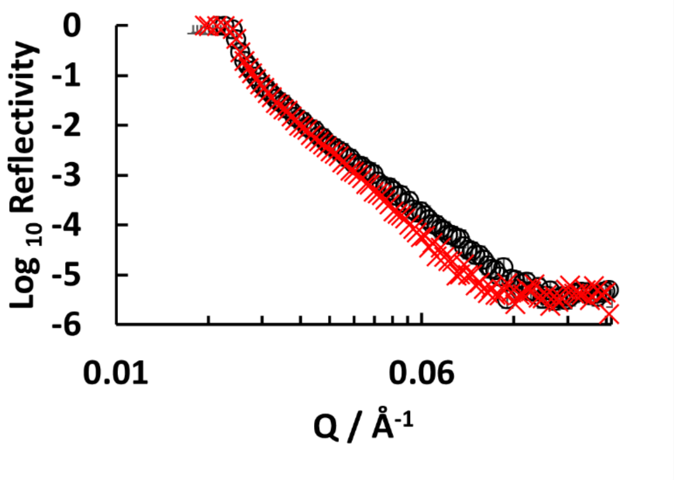

Figure S4. Neutron reflectivity data showing the addition of 0.15 wt. % *Moringa oleifera* seed protein to the cleaned silica 2 surface in (o) D_2O, (x) H_2O. The solid lines show a model fit with the parameters shown in Table S3.

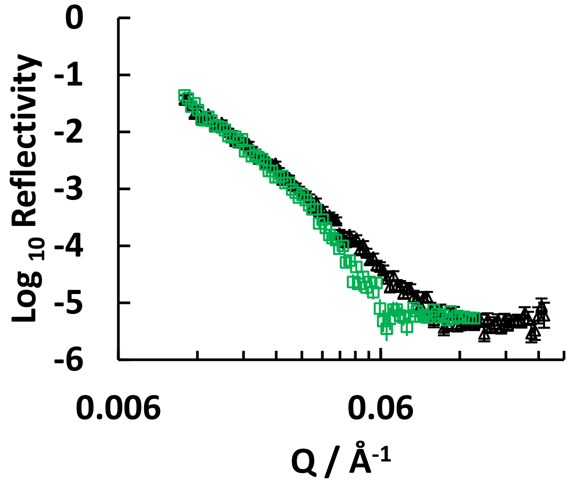

Figure S5. Change in neutron reflectivity data after the addition of (□) 18 mmol dm^{-3} sodium PFOA to pre-adsorbed (Δ) *Moringa oleifera* protein solutions in H_2O.

Table S2. Fitted Parameters for Protein in D_2O

**Surface**	**ρ / 10^{-6} Å^{-2}**	***t*$_p$ / Å**	***l* / Å**	**ϕ_P**	**Γ / mg m^{-2}**	**Γ / µmol m^{-2}**
Silica 1	4.61 ± 0.01	6 ± 1	15 ± 1	0.46 ± 0.01	1.3 ± 0.1	0.2 ± 0.02
Silica 2	4.54 ± 0.01	13.8 ± 0.1	31 ± 1	0.48 ± 0.01	2.9 ± 0.1	0.4 ± 0.03
Silica 1 (after surfactant)	4.61 ± 0.01	13.7 ± 0.1	23 ± 1	0.46 ± 0.01	1.5 ± 0.1	0.2 ± 0.02

t_p = uniform layer thickness, ρ = fitted scattering length density for the uniform layer *l* = Exponential decay length, ϕ_P = volume fraction of protein in the layer.

Note: The first silicon surface had a combined fit in three contrasts D_2O, H_2O and FMW while silica 2 had a combined fit in two contrasts D_2O and H_2O. The only difference in these contrasts was the value of ρ.

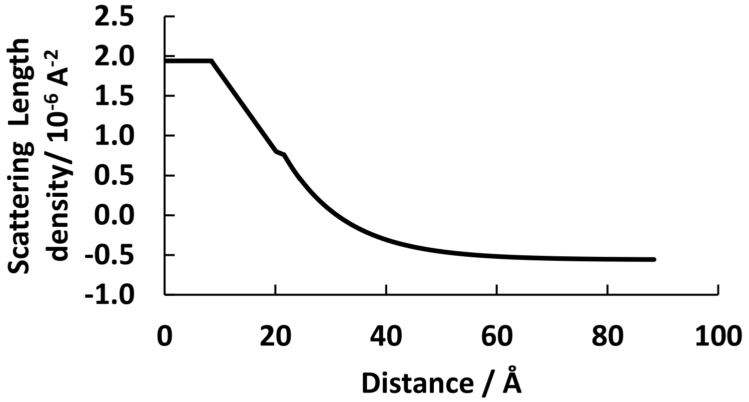

Figure S6. Density profile showing the exponential decay model fit of the adsorbed protein onto the hydrated oxide layer of the silicon surface in H_2O.

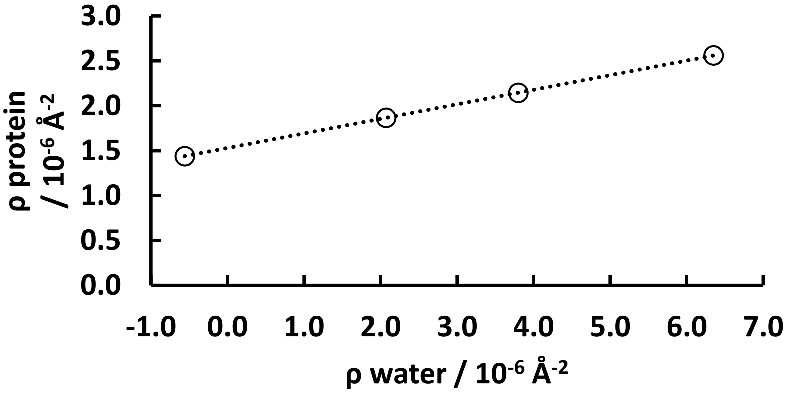

Figure S7. Plot showing the calculated scattering length densities of protein in different water contrasts. The values were calculated from the scattering length densities of protein in H_2O and D_2O found in table 1. Additional values for the other solutions were determined from the straight-line interpolation.

Effect of perfluorooctanoate at different concentrations on Silica 1

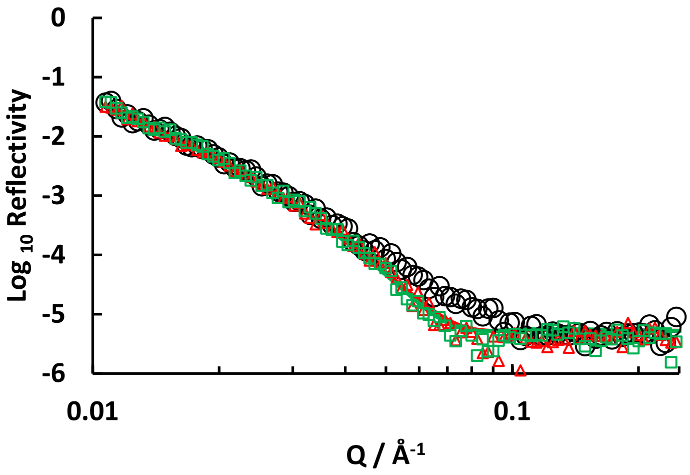

Figure S8. Changes in neutron reflectivity for perfluorooctanoate at (Δ) 4.5 mmol dm^{-3} and (□) 9 mmol dm^{-3} added to the pre-adsorbed layer of *Moringa oleifera* seed protein (o) to silica 1 in H_2O.

Table S3. Fitted Parameters for the protein layers with surfactant

**Surfactant**	**Concentration/ mmol dm^{-3}**	**ρ / 10^{-6} Å^{-2}**	**t_p / Å**	***l* / Å**	**$\phi_{surfactant}$**	**Γ / mg m^{-2}**	**Γ / μmol m^{-2}**
perfluorooctanoate	4.5	4.74 ± 0.01	21.6 ± 0.1	14.5 ± 0. 1	0.23 ± 0. 1	1.5 ± 0. 1	3.4 ± 0. 1
	9	4.78 ± 0.01	23.7 ± 0. 1	14.1 ± 0. 1	0.23 ± 0. 1	1.5 ± 0. 1	3.5 ± 0. 1
	18	4.87 ± 0.01	22.6 ± 0. 1	19.4 ± 0. 1	0.24 ± 0. 1	1.8 ± 0. 1	4 ± 1
pefluorohexanoate	100	5.27 ± 0.01	55.2 ± 0. 1	37.3 ± 0. 1	0.08 ± 0. 01	1.3 ± 0. 1	4 ± 1

t_p = uniform layer thickness, ρ = fitted scattering length density for the uniform layer *l* = Exponential decay length, ϕ_s = volume fraction of surfactant in the layer.
